# A feasibility study for quantitative assessment of cerebrovascular malformations using flutriciclamide ([^18^F]GE-180) PET/MRI

**DOI:** 10.3389/fmed.2023.1091463

**Published:** 2023-04-05

**Authors:** Sally Ji Who Kim, Janine M. Lupo, Yicheng Chen, Miguel H. Pampaloni, Henry F. VanBrocklin, Jared Narvid, Helen Kim, Youngho Seo

**Affiliations:** ^1^Department of Radiology and Biomedical Imaging, University of California, San Francisco, San Francisco, CA, United States; ^2^Cardiovascular Research Center, Cardiology Division, Massachusetts General Hospital, Harvard Medical School, Boston, MA, United States; ^3^Department of Anesthesia and Perioperative Care, Center for Cerebrovascular Research, University of California, San Francisco, San Francisco, CA, United States

**Keywords:** vascular malformation, CCM, PET/MRI, quantitative susceptibility mapping (QSM), flutriciclamide, GE-180, TSPO, neuroinflammation

## Abstract

**Aim:**

Neuroinflammation plays a key role in both the pathogenesis and the progression of cerebral cavernous malformations (CCM). Flutriciclamide ([^18^F]GE-180) is a translocator protein (TSPO) targeting positron emission tomography (PET) tracer, developed for imaging neuroinflammation. The objectives of this study were to describe characteristics of flutriciclamide uptake in different brain tissue regions in CCM patients compared to controls, and to evaluate flutriciclamide uptake and iron deposition within CCM lesions.

**Materials and methods:**

Five patients with CCM and six controls underwent a 60 or 90 min continuous PET/MRI scan following 315 ± 68.9 MBq flutriciclamide administration. Standardized uptake value (SUV) and standardized uptake value ratio (SUVr) were obtained using the striatum as a pseudo-reference. Quantitative susceptibility maps (QSM) were used to define the location of the vascular malformation and calculate the amount of iron deposition in each lesion.

**Results:**

Increased flutriciclamide uptake was observed in all CCM lesions. The temporal pole demonstrated the highest radiotracer uptake; the paracentral lobule, cuneus and hippocampus exhibited moderate uptake; while the striatum had the lowest uptake, with average SUVs of 0.66, 0.55, 0.63, 0.55, and 0.33 for patient with CCM and 0.57, 0.50, 0.48, 0.42, and 0.32 for controls, respectively. Regional SUVr showed similar trends. The average SUV and QSM values in CCM lesions were 0.58 ± 0.23 g/ml and 0.30 ± 0.10 ppm. SUVs and QSM were positively correlated in CCM lesions (*r* = 0.53, *p* = 0.03).

**Conclusion:**

The distribution of flutriciclamide ([^18^F]GE-180) in the human brain and CCM lesions demonstrated the potential of this TSPO PET tracer as a marker of neuroinflammation that may be relevant for characterizing CCM disease progression along with QSM.

## Introduction

Cerebral cavernous malformations (CCM) are abnormal clusters of leaky, enlarged capillaries in the brain or spinal cord that can occur as a sporadic or inherited rare disease in approximately 0.5% of the population (1–4). Approximately 75% of patients with these cerebrovascular malformations have a high risk of intracranial hemorrhages, seizures, focal neurological deficits, and severe headaches secondary to CCM (5). Currently, surgery or radiosurgery of are possible interventional treatment options for patients with symptomatic CCM (6). Although genetic mutations in *KRIT1/CCM1*, *CCM2*, and *PDCD10/CCM3* are known causes of CCM (7), factors influencing rates of disease severity and progression of CCM remain unknown. Quantitative parameters are needed to better understand the evolution of CCM and predict the onset of symptoms for early intervention.

In recent studies, neuroinflammation has been raised as a novel marker of CCM-related vascular injury (1, 2, 8, 9) along with the number, size, and location of CCM lesions. Repetitive microhemorrhages induce lesion malformation growth and attract macrophages and other inflammatory cells for vascular repair. It has been shown that inflammation is highly correlated with the rupture of the malformation (2). Aside from macrophages, there is also significant evidence of other immune cells (polymorphonuclear cells, macrophages, antibodies producing B-lymphocytes, and plasma cells) inside the CCM lesion (10–13). Macrophages were also found to be present in newly formed CCM lesions in transgenic mice (14).

Translocator protein (TSPO) is a transmembrane protein located in the outer membrane of the mitochondria in the central and peripheral nervous system (15–18). For more than three decades, *in vivo* TSPO positron emission tomography (PET) imaging has been widely applied for detecting brain neuroinflammatory processes and associated microglial disease, with limited success initially using earlier TSPO agents (15, 18–24). The third generation of TSPO receptor-ligand for PET imaging, flutriciclamide ([^18^F]GE-180), has improved affinity, permeability, and target to background contrast (25–27) with a longer half-life (109.7 min) than that of ^11^C (20.4 min), which has been used extensively for TSPO PET imaging previously. Flutriciclamide has shown promise as a biomarker for multiple sclerosis, glioma, and other neuroinflammatory diseases, with significant uptake in the target lesion (24, 28–39). By taking advantage of the low expression of TSPO in the brain under normal physiologic conditions, activation of microglial cells caused by inflammatory stimuli results in significant upregulation of TSPO expression (19, 40, 41).

The purpose of this study was to quantify flutriciclamide’s uptake patterns in the brains of control subjects and patients with CCM using TSPO PET and compare these uptake levels caused by neuroinflammation with iron deposition assessed *via* quantitative susceptibility mapping (QSM) (22, 42–44). We hypothesize that the 18 kDa TSPO PET would identify biologically active regions of CCM lesions due to microglia activation and the presence of macrophages that would correlate with iron deposition.

## Materials and methods

### CCM participants and controls

PET/MR imaging was performed on five patients with cerebral cavernous malformations (CCM) and six control participants with human immunodeficiency virus (HIV). Eligible participants for this imaging study were age 18 or older. Patients were excluded from the study if they (1) had a contraindication to the administration of a radiotracer or gadolinium MRI contrast, or (2) were women who were pregnant or lactating at the time of screening due to increased potential risk. All patients provided informed consent to enroll in this study that was approved by our institutional review board.

### Genotyping

A known functional polymorphism of TSPO (Ala147Thr polymorphism) affects binding of several high-affinity TSPO radioligands. The TSPO genotype is comprised of high-affinity binders (HAB), mixed-affinity binders (MAB), and low-affinity binders (LAB). Thus, all participants were genotyped and those with the LAB genotype were additionally excluded. Blood or saliva was collected from enrolled participants for TSPO genotyping. DNA was extracted from blood samples using a Qiagen QIAmp DNA blood mini kit or from saliva samples using Oragene kits at UCSF laboratories using standard protocols. Genotyping was performed using a TaqMan allelic discrimination assay (Applied Biosystems C__2512465_20) and control samples of all three genotypes (HAB, MAB, and LAB) were included in each run.

### Synthesis of flutriciclamide

Flutriciclamide was prepared for human use under a protocol approved by the local radioactive drug research committee (RDRC). Flutriciclamide was synthesized in the Neptis^®^ automated synthesizer in our current good manufacturing practice (cGMP) radiopharmaceutical facility using a commercially available mesylate precursor (GE Healthcare, UK) (45). The flutriciclamide was purified by high performance liquid chromatography, concentrated, and filtered into sterile product vials. The final product passed all quality testing before being released for injection.

### PET/MR imaging acquisition and reconstruction

Participants underwent 60- or 90- min long (30) simultaneous PET and MR scans in a hybrid PET/MRI scanner (SIGNA PET/MR, GE Healthcare, Waukesha, WI, USA) equipped with an 8-channel head coil array. Single intravenous bolus injections of 315 ± 68.9 MBq of flutriciclamide ([^18^F]GE-180) were administered to all participants. Multiple clinical MR sequences including gradient- and spin-echo T1-weighted images pre- and post-gadolinium contrast injection, diffusion weighted imaging (DWI), arterial spin labeling (ASL), MR-angiography, in-phase T1, and a T2*-weighted, multi-echo susceptibility-weighted angiography (SWAN) scan (6 echoes with TEs = 13, 17.3, 21.6, 25.9, 30.2, 34.5 ms, TR = 50.7 ms, flip angle = 20°, FOV = 24 cm, ARC with R = 2 and a reconstructed image matrix of 512 × 512 × 108 with the voxel size of 0.47 mm× 0.47 mm× 1.50 mm) that was used for QSM map generation. A manufacturer-provided time-of-flight-enabled [approximately 400 ps (33)] iterative reconstruction algorithm (28 subsets, 5 iterations, 2 mm full-width at half-maximum post-reconstruction filter) was used for PET image reconstruction, resulting in a volumetric matrix of 256 × 256 × 89 with the voxel size of 1.17 mm× 1.17 mm× 2.78 mm. Atlas-based attenuation correction was applied to the PET images.

### PET image processing and regional analyses

As flutriciclamide uptake plateaus at 60 min (46), all brain PET images were summed over 50–60 min or 60–90 min when the total scan duration was 60 min (29, 30) or 90 min, respectively, to generate consistent standardized uptake values (SUVs) (29, 30, 46–48) between control and CCM participants. The difference in the total scan time (60 min vs. 90 min) was due to logistical issues that truncated the total scan time in a subset of exams. To compare regional SUVs of the tracer in different brain regions between the CCM and control subjects, each static PET image was co-registered and realigned to an automated anatomical labeling (AAL)-region of interest (ROI) atlas using PMOD (version 3.3, PMOD Technologies, Zurich, Switzerland). Each transformation matrix from an in-phase T1 MR to a T1 MR template was used for PET normalization. We chose the striatum as a pseudo-reference region for the evaluation of the SUV ratio (SUVr) in the absence of any true non-specific reference region for TSPO binding (33, 46). Differences in flutriciclamide uptake between control and CCM participant groups were analyzed using unpaired *t*-test, and a *p*-value < 0.05 was considered statistically significant.

### QSM generation and lesion-specific value

The reconstruction pipeline for the multi-echo QSM is as follows. Missing phase-encoding lines were filled in using the auto-calibrating reconstruction for Cartesian sampling (ARC) method for each individual coil (49) and then a channel-wise inverse Fourier transform was applied to obtain the coil magnitude and phase images. MCPC-3D-S (50) was applied to obtain combined magnitude and raw phase images from each coil for every echo. Skull stripping and brain mask extraction were applied to the magnitude images from each echo using FMRIB Software Library (FSL) Brain Extraction Tool (BET) (51) and the final brain mask was generated by calculating the intersection of all the masks. 3D Laplacian phase unwrapping (52) was subsequently applied on the masked phase images to create unwrapped phase images for QSM processing. Sophisticated Harmonic Artifact Reduction for Phase data with varying spherical kernel (VSHARP) (53, 54) was applied on the unwrapped phase images using the STI Suite toolbox in Matlab to remove the background field before performing the dipole inversion step with iLSQR that generated the final QSM maps (55).

### CCM lesion-specific quantification

Cerebral cavernous malformations lesion location, lesion-specific flutriciclamide uptake, and the amount of iron deposition within each lesion were quantified from patients with CCM. CCM ROIs were manually delineated on QSM as hyperintense regions (46, 56) greater than 5 mm in diameter. QSM maps were then resampled to the resolution of the co-registered PET data in order to compare mean SUV to mean QSM value within each CCM lesion.

### Statistical analysis

The GraphPad Prism was used for all statistical analyses. The unpaired *t*-test (*p* < 0.05) of normal non-CCM brain regions in both the CCM patient and control groups was performed. The mean flutriciclamide SUV and mean QSM value in all 17 CCM lesions were compared using the Pearson correlation.

## Results

### Patient characteristics

The average age of the CCM participant group (four males and one female) was 48 ± 15, while the average age of the control group (six males) was 57 ± 5. Among the CCM group, both 60 min (*n* = 2) and 90 min (*n* = 3) PET scans were acquired. Among the controls, both 60 min (*n* = 1) and 90 min (*n* = 5) PET scans were acquired. Four patients with CCM had multiple lesions, while one patient had a sporadic CCM. Patients presented with either focal deficits (*n* = 3), acute hemorrhage (*n* = 1) or seizure (*n* = 1). Three of the patients with multiple CCM had a surgical resection of hemorrhagic lesion prior to the study.

### Tracer uptake patterns in different brain regions

**FIGURE 1:**
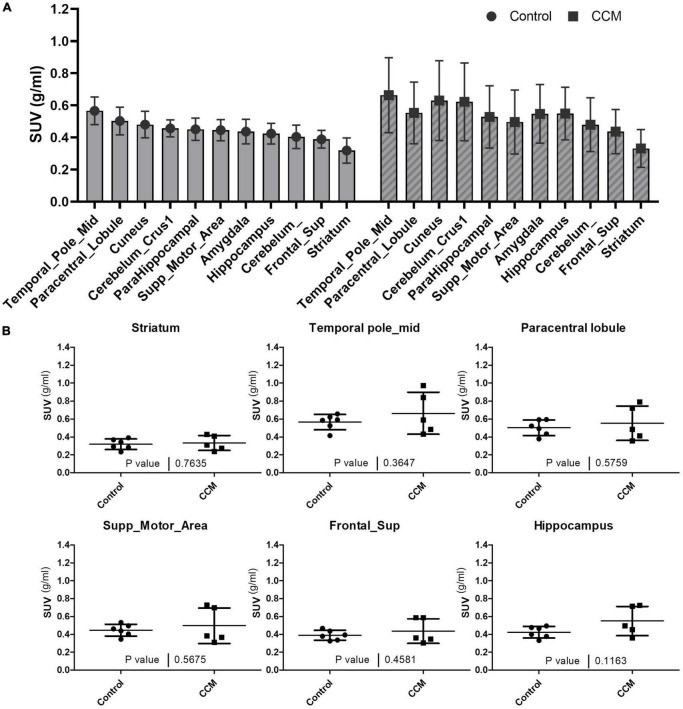
**(A)** Average SUVs with standard deviation of global brain tissue flutriciclamide uptake in control (gray) and CCM groups (striped). The temporal pole indicates the highest uptake, and the striatum indicates the lowest uptake among all the brain tissue regions. **(B)** Comparison of flutriciclamide uptake in representative brain tissue of control and CCM groups. The AAL template was used as a predefined ROI. *P* < 0.05.

For all participants, the visual distribution of flutriciclamide in brain tissue was found to be globally low, with hotspots denoting specific CCM lesion-uptake of flutriciclamide. The temporal pole demonstrated the highest radiotracer uptake (mean SUV = 0.66 ± 0.23); the paracentral lobule, cuneus, and hippocampus exhibited moderate uptake (0.55 ± 0.19, 0.63 ± 0.25, 0.55 ± 0.16, and, respectively); and the striatum had the lowest uptake 0.33 ± 0.12 in the CCM cohort, as indicated in Figure 1A and Table 1. The regional pseudo-SUVr measurements followed a similar trend (Table 2). Although the controls consistently exhibited reduced SUV across all brain regions, the same trends existed between regions for both SUV and SUVr, with the temporal pole likewise demonstrating the highest radiotracer uptake, followed by the paracentral lobule, cuneus, and hippocampus exhibiting moderate uptake, and the striatum having the lowest uptake (average SUVs of 0.57 ± 0.09, 0.50 ± 0.09, 0.48 ± 0.08, 0.42 ± 0.06 and 0.32 ± 0.08, respectively; Table 1). Similarly, regional pseudo-SUVr’s were 1.81 ± 0.34, 1.59 ± 0.34, 1.53 ± 0.24, and 1.34 ± 0.16, respectively (Table 2). The global distribution of SUVs between patients with CCM and controls were within a similar range, with no statistically significant differences found between groups in all normal brain regions analyzed, as illustrated in Figure 1B. All non-CCM brain tissue comparisons between the two groups excluded CCM lesions, hemorrhage, and surgery areas.

**TABLE 1:** Regional flutriciclamide SUVs (g/ml) in the global brain tissue region for both the CCM and control group.

Non-CCM ROI	CCM	Control
Temporal Pole_Mid	0.66 ± 0.23	0.57 ± 0.09
Cuneus	0.63 ± 0.25	0.48 ± 0.08
Paracentral lobule	0.55 ± 0.19	0.50 ± 0.09
Calcarine	0.56 ± 0.21	0.44 ± 0.05
Cerebellum_Crust1	0.62 ± 0.24	0.46 ± 0.05
Supp_Motor _Area	0.50 ± 0.20	0.45 ± 0.07
Vermis (1–10)	0.49 ± 0.19	0.37 ± 0.07
Amygdala	0.55 ± 0.18	0.44 ± 0.08
Frontal_Sup	0.44 ± 0.14	0.39 ± 0.06
Striatum	0.33 ± 0.12	0.32 ± 0.08
Hippocampus	0.55 ± 0.16	0.42 ± 0.06
Cingulum_Post	0.47 ± 0.15	0.41 ± 0.09
Precuneus	0.54 ± 0.19	0.44 ± 0.07
Cerebellum (3–10)	0.48 ± 0.17	0.40 ± 0.07
ParaHippocampal	0.53 ± 0.19	0.45 ± 0.07
Occipital Sup	0.51 ± 0.17	0.43 ± 0.07

All regions-of-interest (ROIs) are based on the AAL template. The striatum consists of caudate, and putamen, which are defined in the AAL. The temporal pole and striatum consistently show the highest and lowest uptake in both groups.

**TABLE 2:** Regional pseudo-SUVr of flutriciclamide in brain tissue regions for both the CCM and control group.

Non-CCM ROI	CCM	Control
Temporal pole	1.96 ± 0.25	1.81 ± 0.34
Cuneus	1.85 ± 0.32	1.53 ± 0.24
Paracentral lobule	1.64 ± 0.24	1.59 ± 0.23
Calcarine	1.64 ± 0.21	1.41 ± 0.13
Cerebellum_Crus1	1.83 ± 0.29	1.47 ± 0.28
Supp motor area	1.46 ± 0.25	1.42 ± 0.17
Vermis (1–10)	1.44 ± 0.28	1.19 ± 0.22
Amygdala	1.63 ± 0.22	1.39 ± 0.21
Frontal_Sup	1.30 ± 0.11	1.26 ± 0.12
Hippocampus	1.64 ± 0.16	1.34 ± 0.16
Cingulum_Post	1.41 ± 0.23	1.30 ± 0.09
Precuneus	1.60 ± 0.22	1.39 ± 0.14
Cerebellum (3–10)	1.42 ± 0.24	1.28 ± 0.18
Parahippocampus	1.56 ± 0.19	1.43 ± 0.15
Occipital sup	1.52 ± 0.15	1.36 ± 0.21

The striatum was selected as a pseudo-reference for SUVr.

### Tracer uptake and iron deposition within CCM lesions

**FIGURE 2:**
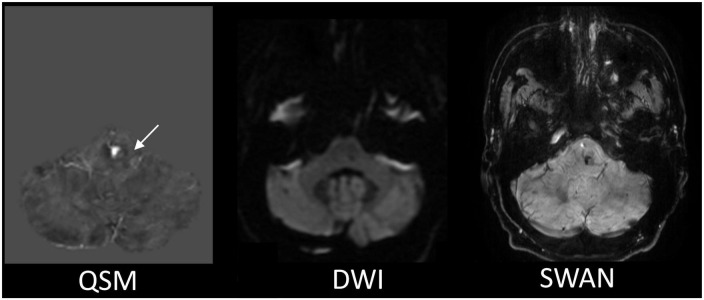
The representative MR images comparison for the CCM detection. The QSM displays the most apparent boundary of the CCM from among all the MR images we acquired. The white arrow is pointing to the location of the CCM.

**FIGURE 3:**
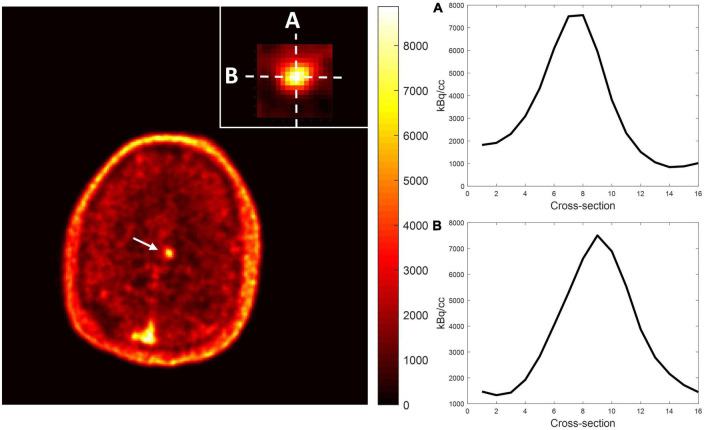
Representative image contrast of flutriciclamide PET across the CCM lesion. Display vertical **(A)** and horizontal **(B)** cross-sections of CCM and nearby brain tissue areas.

**FIGURE 4:**
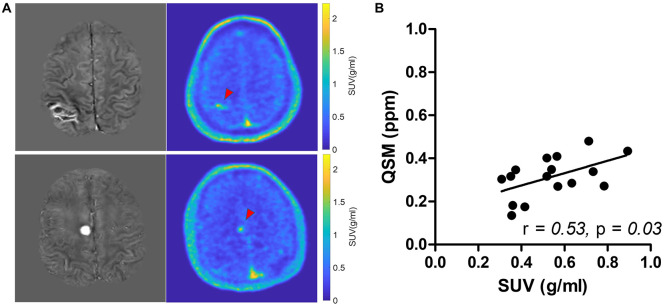
**(A)** Comparison of QSM (left) and SUV map of flutriciclamide (right) of two representative cerebral cavernous malformations (CCM) cases. The red arrow points at the CCM lesion in the brain. **(B)** Correlation of flutriciclamide SUV and QSM in CCM lesion.

**FIGURE 5:**
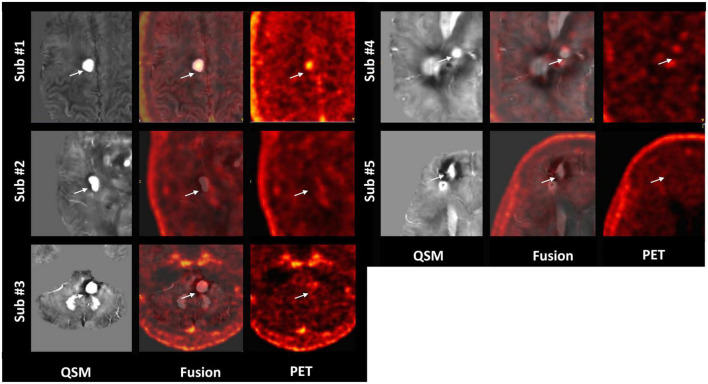
Representative QSM and matched flutriciclamide PET images demonstrate the heterogeneity of the CCM lesions for all CCM patients. The white arrow is pointing to the location of the CCM.

Among the multiple acquired MR sequences, QSM demonstrated the highest level of sensitivity for CCM (Figure 2). Average CCM lesion sizes measured on QSM ranged from 68.0 mm^3^ to 2.3 cm^3^. CCM lesion location and each patient’s average within-lesion flutriciclamide SUVs are presented in Table 3. The average flutriciclamide SUV and QSM values were 0.58 ± 0.23 g/ml and 0.30 ± 0.10 ppm, respectively, among 17 CCM lesions. The average image contrast ratio of the flutriciclamide uptake in the CCM lesion over the remainder of the non-CCM brain tissue region in the vicinity was 6-7-fold high, as observed in the cross-section profile in Figure 3. Figure 4A shows the increased flutriciclamide uptake within two CCM lesions. Qualitatively, flutriciclamide uptake in CCM lesions was heterogeneous while CCM lesions were overall larger and could appear either heterogenous or uniformly hyperintense on QSM (Figures 4A, 5). SUV of flutriciclamide was positively correlated with QSM values within CCM lesions (*r* = 0.53, *p* = 0.03) as indicated in Figure 4B.

**TABLE 3:** Each CCM subject’s lesion type, CCM location, flutriciclamide uptake, and SUVs.

Case #, gender	Type of CCM	Lesion location	Lesion SUV (g/ml) average ± *S.D
Subject #1, M	Multiple (*n* = 6)	Cerebellum_9_R, Pons, Fusiform_R, Caudate_R, Corpus Callosum, Supp_Motor_Area_R	0.50 ± 0.15
Subject #2, F	Multiple (*n* = 4)	Cuneus_R, Tail of Caudate Nucleus, Temporal_Sup_R, Frontal_Sup_Orb_R	0.57 ± 0.16
Subject #3, M	Sporadic (*n* = 1)	Cerebelum_4_5_L, Cerebelum_6_L	0.52
Subject #4, M	Multiple (*n* = 3)	Temporal_Sup_R, Temporal_Mid_R, Temporal_Inf_R, Post-central_R, Precuneus_L	0.38 ± 0.03
Subject #5, M	Multiple (*n* = 3)	Pons, Temporal_Mid_L, Frontal_Sup_R, Frontal_Sup_Orb_R, Frontal_Mid_Orb_R, Frontal_Mid_R	0.95 ± 0.21
Average	(*n* = 17)		0.58 ± 0.23

*S.D denotes standard deviation of the SUV.

## Discussion

Our study aimed to explore neuroinflammation using multimodal TSPO PET/MRI imaging for flutriciclamide quantification in cases with cerebral cavernous malformations. Flutriciclamide uptake indicates the level of neuroinflammation of a particular brain region. Neuroinflammation in the physiology and clinical course of CCM disease has been reported in recent years (1, 2, 57). A CCM-associated proinflammatory cytokine, IL-1 β, was reported to increase endothelial permeability, promoting leukocyte transmigration and resulting in imminent symptomatic lesional bleeding in the brain (58). In addition, the highly inflammatory state was associated with seizure activity, more than one hemorrhagic event, and a higher rate of new CCM formation and lesion growth (2). Therefore, neuroinflammation quantification is promising for diagnosing the risk of CCM rupture and screening patients for brain surgery non-invasively. Our approach fully incorporated the merits of PET/MRI in not only delineating CCM lesion location but also quantifying both neuroinflammation and iron deposition simultaneously. Iron deposition from QSM indicates hemoglobin concentrations in the region, and a threshold increase of 6% or more in QSM has been shown to reflect new symptomatic hemorrhage in previously stable lesions (59). QSM has thus been proposed as a monitoring biomarker for CCM (60). However, neuroinflammation also plays an important role in CCM pathogenesis and severity, with immune cell infiltration (B-lymphocytes and plasma cells) in CCM lesions and abnormally high levels of inflammatory cytokines and immunoglobulins expressed both in tissue and blood of CCM patients (9). Patients with a high inflammatory state based on 4 plasma inflammatory markers had a higher rate of new hemorrhage, lesion growth, or new lesion formation during prospective follow-up (2). Thus, these two imaging indicators, namely, iron deposition and neuroinflammation, may prove useful for identifying lesions at higher risk for rupture. To our knowledge, this is the first study examining the characteristics of flutriciclamide and its applicability for CCM.

We observed that flutriciclamide is a CCM lesion-specific indicator reflecting neuroinflammation in CCMs. The SUVs in various normal brain regions exhibited a range comparable to those reported in other flutriciclamide studies (46). Flutriciclamide uptake was only visible in CCM lesions for the CCM group, and a heterogeneous distribution was observed within lesions (Figures 4A, 5). The SUVs of all lesions were higher than those of normal brain tissue. Although it remains unclear what the most appropriate reference region is for flutriciclamide (31), we chose the striatum as a pseudo-reference region because of its relatively low-TSPO uptake.

Among the multiple MR images we obtained, QSM was the superior imaging technique for delineating CCM lesions (Figure 2). Our results aligned with those of previous studies (3, 21, 61), further substantiating QSM as an important surrogate marker for evaluating lesions in patients with CCM. Both neuroinflammation and iron deposition were observed simultaneously within CCM lesions, resulting in a moderate but significant correlation between these parameters. This suggests that ruptured CCM vasculature results in inflammation. As seen in Figures 4A, 5, the fact that respective distributions of neuroinflammation and iron deposition are spatially distinct, suggests that they provide separate but complementary information for future studies in CCM.

This study has several limitations. First, the small sample size precluded our ability to adequately compare regional differences in tracer uptake between groups. Although a few individual patients with CCM had increased uptake in the temporal pole, paracentral lobule, SMA, and hippocampus, compared to the control group, no definitive conclusions could be made. The small sample size also prevented adjustments or additional subgroup analyses comparing groups categorized by sporadic or familial disease, gender, recurrence rate, year of first hemorrhage, age, or clinical symptoms. In addition, previous studies used different protocols that featured two scanning durations comparing the most similar status at around 60 min (50–60 min and 60–90 min), which is the equilibrium state period (46). Our use of patients with HIV as a control group could also be viewed as a limitation since HIV is an inflammatory disease. However, flutriciclamide did not reflect the potential risk as a neuroinflammation reservoir, possibly because TSPO binding, or expression is disease-specific (18). Despite these limitations, this study still provided evidence that warrants future studies with this tracer in a larger population of patients with CCM.

In conclusion, flutriciclamide, a TSPO imaging tracer, is a potential marker of neuroinflammation in CCM and may detect CCM lesions with adequate contrast. When combined with QSM for lesion delineation, complementary information is obtained spatially, demonstrating lesion heterogeneity. This combined TSPO PET/MRI scanning approach could be used for longitudinal monitoring of disease progression and evaluating the effectiveness of potential drug therapies in future clinical trials.

## Data availability statement

The volunteer datasets generated for this study are available upon request to the corresponding author.

## Ethics statement

The studies involving human participants were reviewed and approved by the University of California, San Francisco (UCSF) IRB. The patients/participants provided their written informed consent to participate in this study. All procedures performed in studies involving human participants were in accordance with the ethical standards of the local Institutional Review Board (IRB) and with the 1964 Helsinki Declaration and its later amendments or comparable ethical standards.

## Author contributions

SK: data analysis, concept, design of study, and manuscript writing. JL: design of study, contribution to a specific section of the manuscript, and proofreading. YC: data generation. MP: concept and data acquisition. HV: tracer production and proofreading. JN: data acquisition. HK: concept, design of study, data acquisition, and proofreading. YS: concept, design of study, and proofreading. All authors contributed to the article and approved the submitted version.
